# Effect of nordihydroguaiaretic acid on cell viability and glucose transport in human leukemic cell lines

**DOI:** 10.1002/2211-5463.12106

**Published:** 2016-08-23

**Authors:** David Leon, Daniela Parada, Mauricio Vargas‐Uribe, Alejandra A. Perez, Lorena Ojeda, Angara Zambrano, Alejandro M. Reyes, Mónica Salas

**Affiliations:** ^1^ Facultad de CienciasInstituto de Bioquímica y MicrobiologíaUniversidad Austral de ChileValdiviaChile

**Keywords:** cell viability, glucose transporter GLUT1, leukemic cells, nordihydroguaiaretic acid

## Abstract

The polyphenol nordihydroguaiaretic acid (NDGA) has antineoplastic properties, hence it is critical to understand its action at the molecular level. Here, we establish that NDGA inhibits glucose uptake and cell viability in leukemic HL‐60 and U‐937 cell lines. We monitored hexose uptake using radio‐labeled 2‐deoxyglucose (2DG) and found that the inhibition by NDGA followed a noncompetitive mechanism. In addition, NDGA blocked hexose transport in human red blood cells and displaced prebound cytochalasin B from erythrocyte ghosts, suggesting a direct interaction with the glucose transporter GLUT1. We propose a model for the mechanism of action of NDGA on glucose uptake. Our study shows for the first time that NDGA can act as inhibitor of the glucose transporter GLUT1.

**Abbreviations**
2DG: 2‐deoxyglucose
CCB: cytochalasin B
GLUT1: facilitated glucose transporter member 1
HL‐60: human promyelocytic leukemia cells
LOX: lipoxygenase
NDGA: nordihydroguaiaretic acid
PBMC: peripheral blood mononuclear cells
U‐937: human monoblast leukemia cells

One of the most remarkable metabolic traits of cancer cells is their increased rate of glucose breakdown through glycolysis and lactic acid fermentation, even under aerobic conditions. This phenomenon, called Warburg effect, allows cancer cells to sustain high proliferation rates because it ensures rapid production of ATP and metabolic intermediates to feed biosynthetic pathways 1, 2. Because this metabolic feature demands a high supply of glucose, cancer cells upregulate the expression of GLUT1, a facilitative transporter that enables the diffusion of glucose across the plasma membrane 3, 4. Thus, blocking glucose uptake and its subsequent breakdown through pharmacologic agents is a promising strategy to hamper cell growth by glucose deprivation, and to ultimately cause cell death.

The kinetic properties of GLUT1 have been thoroughly studied in human red blood cells because of the high expression of this carrier in their plasma membrane 5, 6. This has allowed the discovery of small natural and synthetic molecules, with no structural similarity with glucose, that act as inhibitors of GLUT1. Examples of inhibitors of GLUT1 include flavones, isoflavones and tyrphostins, all of which differ in their mechanism of inhibition 7, 8, 9, 10, 11, 12, 13, 14. Recently, we described that resveratrol (RSV), a polyphenol of natural origin with multiple cellular targets, is a direct inhibitor of GLUT1‐mediated glucose transport 15.

Nordihydroguaiaretic acid (NDGA), a polyphenol that shares structural similarity with RSV, has been used in traditional medicine to treat a variety of illnesses, including infertility, arthritis, diabetes, pain, inflammation, among others 16. Several studies have shown that NDGA, indeed, affects a wide variety of physiological processes 17, 18, 19, including cancer cell viability and proliferation 20, 21. Because NDGA shows structural similarity with GLUT1's inhibitor resveratrol (and also to other glucose uptake inhibitors such as quercetin), the antineoplastic properties of this polyphenol may be explained by inhibitory effects on glucose uptake. To our knowledge, however, there is no available information regarding the effect of NDGA on glucose uptake.

Here, we test this hypothesis by monitoring the effect of NDGA on both transport and accumulation of glucose analogs in HL‐60 and U‐937 human leukemic cells. We chose these cell lines because acute myeloid leukemia is a common leukemia in adults and the current chemotherapy requires high dosage of drugs. Our results show that NDGA not only affects cell viability but also decreases both sugar uptake and metabolic accumulation into leukemic cells. We provide evidence and a model for the inhibition of glucose transport by a direct interaction between NDGA and GLUT1.

## Materials and methods

### Materials

d‐glucose, sodium bisulfite, sodium phosphate dibasic anhydrous, sodium chloride, potassium chloride, magnesium chloride, potassium phosphate monobasic, sodium dodecyl sulfate (SDS), HEPES, dimethylsulfoxide, dithiothreitiol, and TRIS‐base were obtained from JT Baker. Cytochalasin B, cytochalasin E, d‐sorbitol, 2‐deoxy‐d‐glucose (2DG), 3‐*O*‐methyl‐d‐glucose (OMG), and nordihydroguaiaretic acid (NDGA) were obtained from Sigma Chemical Co, St. Louis, MO, USA. All radioisotopes (2‐[1,2‐^3^H(*N*)]‐deoxy‐d‐glucose, 36.2 Ci mmol^−1^; 3‐*O*‐[methyl‐^3^H]‐methyl‐d‐glucose, 86.7 Ci mmol^−1^; [4‐^3^H(*N*)]‐cytochalasin B, 20 Ci mmol^−1^) were from American Radiolabeled Chemicals, St. Louis, MO, USA. Trypan Blue, HyClone RPMI 1640 cell culture media and FBS were from Thermo Fisher Inc., Waltham, MA, USA.

### Erythrocyte isolation and preparation of membranes

Human red cells were isolated and alkali‐treated erythrocyte ghosts were prepared as previously described 15. Blood was obtained from units (containing dextrose, adenine, and sodium citrate as anticoagulant) provided by the blood Bank Unit of the Valdivia Regional Hospital. All procedures were approved by the Ethics Committees from the Valdivia Regional Hospital and the Universidad Austral de Chile.

### Cell lines

Human leukemic HL‐60 (ATCC CCL240) and U937 (ATCC CRL1593.2) cells were maintained in culture at 37 °C and 5% CO_2_, in RPMI‐1640 without l‐glutamine and supplemented with 10% FBS, and antibiotics. Cell number and viability were determined in a Neubauer chamber using trypan blue exclusion.

### Viability assay

Human leukemic cells were plated at 2 × 10^5^ cells on 24‐well tissue culture plates and treated with different concentrations of NDGA for the indicated times. Then, 2 × 10^4^ cells were seeded in 96‐well tissue culture plates in the presence of 0.1 mg·mL^−1^ neutral red 22. After 2 h of incubation, cells were washed with PBS buffer to remove unincorporated dye. Absorbance at 540 nm was used for quantification.

### Sugar transport and cytochalasin displacement assays

The protocols for infinite‐cis net sugar efflux experiments (Sen–Widdas assays), zero‐trans uptake assays and cytochalasin B binding to erythrocyte membranes were done as previously described 15.

### Data analysis

Statistical analysis and curve fitting were done with prisma 6.0 (GraphPad Software, Inc., La Jolla, CA, USA). All data are presented as means with error bars (means ± SE). In figures, *n* indicates the number of independent experiment performed. Differences between means were analyzed using Student's *t*‐test for paired or unpaired data, wherever appropriate with a *P* value of < 0.05 taken as statistically significant. Inhibition and saturation data were analyzed by nonlinear regression. Hanes–Wolf plots were used to visually display the results.

## Results

### Effect of NDGA on viability of HL‐60 and U‐937 cells

**Figure 1:**
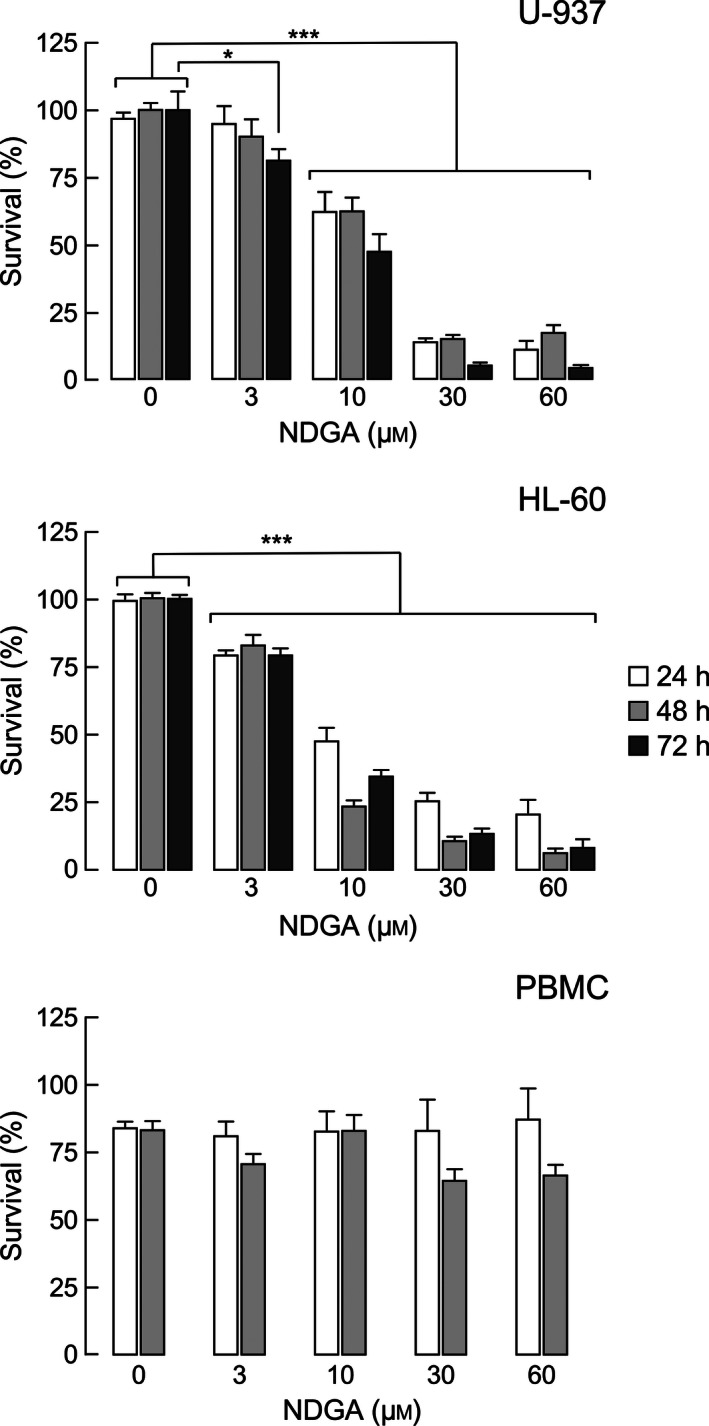
Survival of leukemic cell lines treated with various concentrations of NDGA. Bar graphs represent survival of HL‐60, U‐937, and PBMC cells treated with the indicated NDGA concentrations during 24, 48 or 72 h, respectively. Cell viability was assessed by neutral red assay. The values are expressed as the mean of surviving cells (% of control) ± SEM of four independent experiments performed in triplicate. Significance was determined by a two‐way ANOVA and Bonferroni post‐test. ***P‐value < 0.001; *P‐value > 0.05. For PBMC cells, the statistical analysis was not significant, *P*‐value > 0.05.

Independent studies suggest that NDGA has an important role in proliferation and survival, inducing apoptosis in several human cancer cells 20, 21. As it is unclear whether this holds true for leukemic cell lines, we first tested the effect of NDGA on cell viability of the human leukemic cell lines HL‐60 and U‐937. We treated the cells with various concentrations of NDGA and tested cell viability with neutral red assay, a colorimetric assay that monitors the ability of cells of incorporating dye into lysosomes 22. Figure 1 shows that the incubation of these cell lines with NDGA decreases cell viability in a dose‐dependent manner, achieving nearly full inhibition of viability at 30 μm of NDGA. Incubation for 24, 48, and 72 h resulted in similar readings, indicating that 24 h of incubation is sufficient to observe an effect. Table 1 summarizes the determined IC_50_ values for both cell lines.

**Table 1:** IC_50_ values for the effect of NDGA on cell viability in HL‐60 and U‐937 cell lines

Cell line	IC_50_ at 24 h (μm)	IC_50_ at 48 h (μm)	IC_50_ at 72 h (μm)
HL‐60	10.3 ± 1.7	5.8 ± 0.5	6.7 ± 0.4
U‐937	12.5 ± 2.9	9.9 ± 3.4	7.5 ± 1.0

Cell viability assessed after the treatment of 2.0 × 10^6^ cells with micromolar concentrations of NDGA for 24, 48, and 72 h by neutral red assay and expressed as % of control (survival without NDGA). Data are expressed as mean ± SEM of four independent experiments performed in triplicate.

To assess if the effect of NDGA was specific for exponentially growing cells, we performed the experiment with peripheral blood mononuclear cells (PBMC). PBMC are blood cells with slow proliferation rate, so they serve as a model for slow‐proliferating normal cells and as a control for leukemic cells. NDGA did not impact PBMC viability at doses and incubation times that acutely affected the leukemic cell lines (Fig. 1). These results indicate that highly proliferating leukemic cells are more prone to NDGA action, suggesting that it perturbs one or more cellular processes involved in cell viability and proliferation.

### Effect of NDGA on 2DG transport

**Figure 2:**
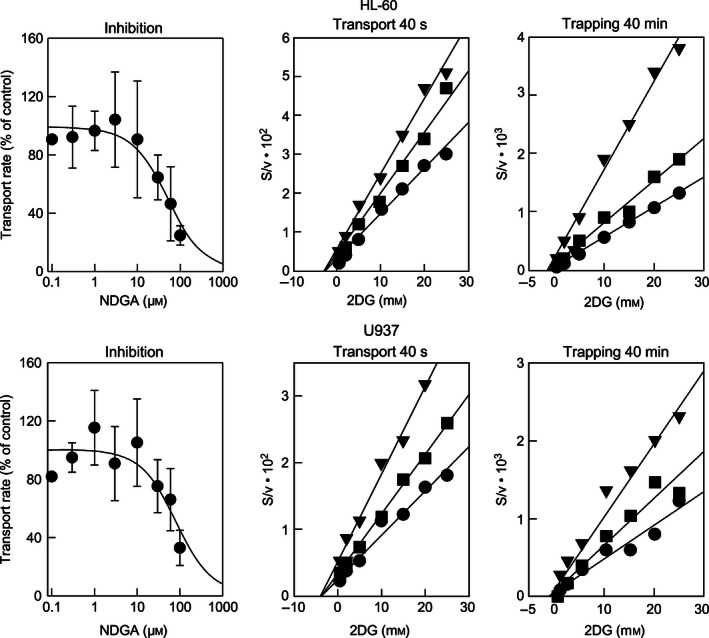
Effect of NDGA on 2DG transport and trapping in HL‐60 cells (upper panels) and U937 cells (lower panels). Inhibition panels correspond to experiments in which transport of 0.25 mm 2DG was monitored in the presence of the indicated NDGA concentrations (*n* = 4). IC_50_ values were obtained by nonlinear fitting of the data to a 1‐parameter hyperbolic inhibition model (solid lines). Transport panels correspond to Hanes–Woolf plots of 2DG saturation curves done in the absence (●) or in the presence of 30 (■) or 60 μm (▼) NDGA in U‐937 and HL‐60 cells, respectively (*n* = 4). Trapping panels correspond to Hanes–Woolf plots of 2DG saturation curves for trapping (40‐min assays) done in the absence (●) or in the presence of 60 (■) or 120 μm (▼) NDGA (*n* = 4) in U‐937 and HL‐60 cells, respectively. In Hanes–Woolf plots, solid lines correspond to linear fits of the data, and a common intercept in the abscissa and increasing slopes are indicative of noncompetitive inhibition. Data are shown as mean ± SD.

Previous research indicated that the time course of 2‐deoxyglucose (2DG) uptake has two components: a fast and linear component (< 5‐min), which represents 2DG membrane transport (2DG transport); and a second linear component (> 5‐min), which accounts for phosphorylation and intracellular accumulation of phosphorylated 2DG (2DG trapping) 15. Distinguishing between transport and intracellular accumulation is critical for proper interpretation of the data. Thus, we carried out uptake assays upon short (40 s) and long (40 min) incubation times to test 2DG transport and trapping, respectively. Figure 2 shows representative assays for the inhibition of 2DG transport in *zero‐trans* entry conditions by various concentrations of NDGA in U‐937 and HL‐60 cells. NDGA inhibits 2DG transport in a dose‐dependent manner with IC_50_ values of 85 and 53 μm for HL‐60 and U‐937 cells, respectively. Under trapping conditions, NDGA also decreased 2DG entry in a dose‐dependent manner, with IC_50_ values of 89 and 103 μm for HL‐60 and U‐937 cells, respectively (data not shown). These IC_50_ values are about 5–10 times higher than those observed for the inhibition of cell viability (see Discussion).

Hexose transport in HL‐60 and U937 cells is functionally coherent with the fact that GLUT1 is the predominant GLUT carrier in these cellular models 23, 24. To reveal the nature of the interaction of NDGA with GLUT1, we performed transport assays using increasing concentrations of 2DG under *zero‐trans* entry conditions in the presence of several fixed NDGA levels (Fig. 2, transport). The observed common intercepts on the *x*‐axis (equal to −*K*_M_) and increasing slopes (equal to 1/*V*_max_) in Hanes–Woolf plots of the transport data are consistent with a noncompetitive mechanism of inhibition, as there is no effect over *K*_M_, but *V*_max_ is decreased. In 2DG trapping assays (Fig. 2, trapping), we observed the same behavior: common intercept on the *x*‐axis and increasing slopes as a function of NDGA concentration in Hannes–Woolf plots. Thus, we concluded that under *zero‐trans* entry conditions NDGA inhibits both 2DG transport and trapping in a noncompetitive manner in HL‐60 and U‐937 cell lines.

**Figure 3:**
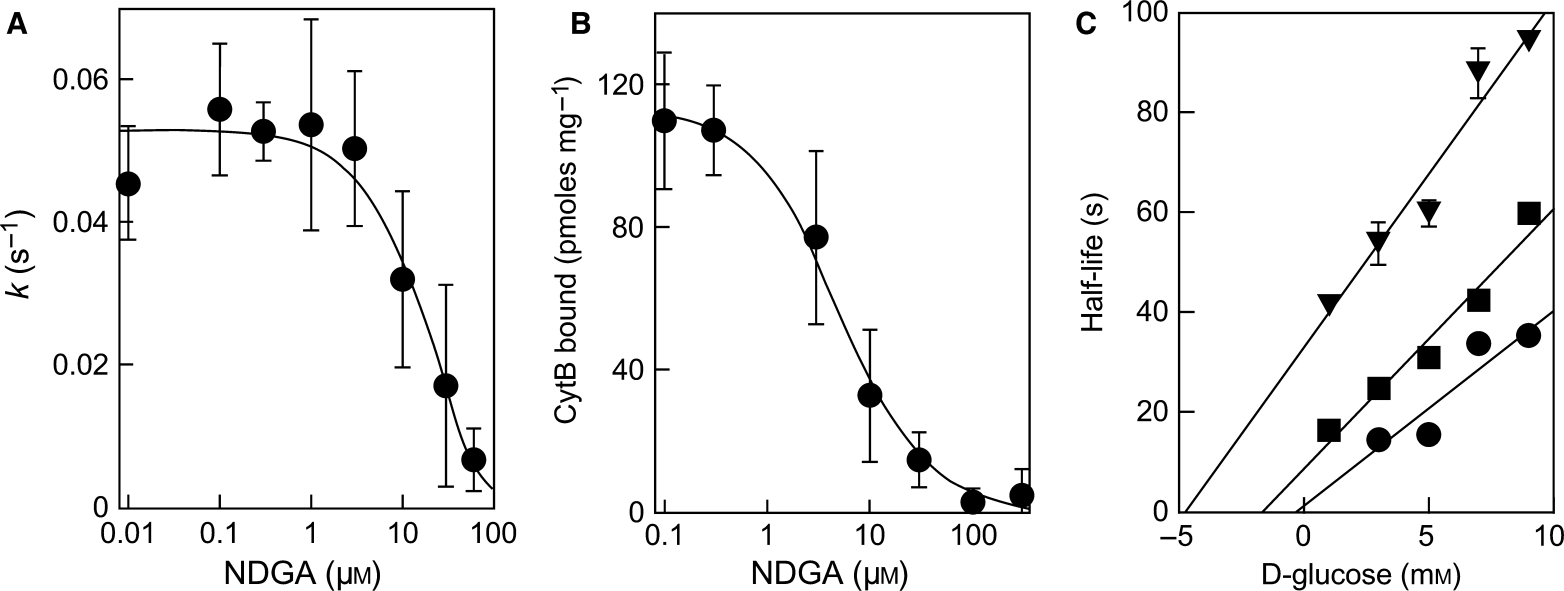
NDGA interaction with GLUT1 in human erythrocytes. Panel A: dose dependence of the inhibition of net d‐glucose exit rate from human erythrocytes by NDGA. The solid line represents the nonlinear fit of the data to a 1‐parameter hyperbolic inhibition model with an IC_50_ value of 26 ± 13 μm (*n* = 4). Panel B: NDGA displacement of the d‐glucose‐displaceable cythocalasin‐B bound to the glucose transporter GLUT1 in human erythrocyte ghosts. The solid line represents the nonlinear fitting of the data to a 1‐parameter hyperbolic inhibition model with a *K*_D__app_ of 4.5 μm (*n* = 1). Panel C: Sen–Widdas plot of the half‐time of d‐glucose exit at different concentrations of d‐glucose in the external medium in the absence (●) or in the presence of 7.5 (■) or 15 μm (▼) NDGA (*n* = 5). Solid lines correspond to linear regression fit for each curve. All data are shown as mean ± SD.

To confirm whether NDGA indeed inhibits the bidirectional carrier GLUT1, we monitored glucose exit in human red blood cells. GLUT1 constitutes the main glucose transporter and near 2–3% of the total membrane protein in this experimental system 5. Under *zero‐trans* exit conditions, NDGA decreased d‐glucose exit in a dose‐dependent manner, with IC_50_ value of about 26 μm (Fig. 3A), which is only about twice the IC_50_ value determined for the inhibition of viability of human leukemic cell lines. Inhibition of transport on human red blood cells indicates that NDGA affects the function of the glucose transporter GLUT1.

### Direct interaction of NDGA with GLUT1

Next, we performed a cytochalasin B (CCB) displacement assay with purified human erythrocyte ghosts to test the direct interaction of NDGA with the transporter. This assay has been previously used to test interactions of small molecules with GLUT1, as binding of CCB to the transporter is affected by that of other molecules 9, 10, 11, 12, 13, 14, 15. Figure 3B shows that increasing concentrations of NDGA displaced previously bound CCB from purified erythrocyte ghosts with *K*_Dapp_ of 4.5 μm. These results indicate that the polyphenol exerts its inhibitory effect by interacting directly with GLUT1.

There are two binding sites for d‐glucose described in the transporter: one facing the extracellular compartment (external site) and one facing the cytosol (internal site). The effect of NDGA on the external binding site can be directly assessed if transport is measured under *cis‐infinite* exit conditions (exit of preloaded d‐glucose in the presence of extracellular concentrations of d‐glucose) with different concentrations of extracellular d‐glucose and inhibitor 25. Figure 3C shows the results arranged as a Sen–Widdas plot to directly visualize changes in dissociation constant of the hexose for the external binding site. The data suggest that NDGA affects the affinity of d‐glucose for the external site, as judged by the displacement of the intercept along the *x*‐axis in the presence of NDGA, while it does not affect the *V*_max_ for d‐glucose exit (notice the similar slopes). These results provide persuasive evidence that NDGA competes with the external d‐glucose‐binding site on the GLUT1 carrier.

## Discussion

NDGA is a polyphenol of natural origin that causes multiple cellular and physiological effects, including blocking tumor growth in animal models. NDGA is a potential candidate to complement cancer therapies; however, the mechanism of action is unclear. As most proliferating undifferentiated cells, including leukemic cells, rely heavily on glucose catabolism to supply their energy needs 4, we investigated if NDGA could indeed affect cellular glucose uptake. Specifically, we addressed the following questions: Given the structural similarity with resveratrol and quercetin, which are known inhibitors of glucose uptake, does NDGA inhibit glucose transporters? Does NDGA inhibit cell viability of human leukemic cell lines? and, is there any correlation between the inhibition of glucose uptake and cell viability in leukemic cells?

Our data indicate that NDGA inhibits and interacts directly with the glucose transporter GLUT1. First, we observed a single saturable component on our experimental system with *K*_M_ values consistent with the functional properties of GLUT1 5, 26. As it is known that the main glucose transporter in the plasma membrane of HL‐60 and U937 cell lines is, indeed, GLUT1 23, 24, the inhibition by NDGA may be the result of inhibiting this carrier. Second, we detected the inhibition of glucose uptake in red blood cells, a cellular system widely used to characterize GLUT1 due of its high expression levels on the cell surface 5, 6, 26. Third, we demonstrated that NDGA displaced CCB previously bound to red blood cell ghosts. CCB is a well‐known GLUT1 inhibitor that binds to the internal face of the transporter, whose displacement serves to judge whether other molecules directly interact with the transporter. Note that NDGA displaces prebound CCB from the carrier at lower concentrations than that required for achieving its inhibitory effect over glucose transport, indicating that the binding of the inhibitor to the transporter does not result in its immediate inhibition unless NDGA concentration is much higher.

To better understand the inhibitory mechanism of NDGA on glucose transport, we performed two types of kinetic tests: 2DG transport *zero‐trans* entry experiments performed in leukemic cells, and d‐glucose *infinite‐cis* exit experiments in human red blood cells. NDGA behaves as a noncompetitive inhibitor in *zero‐trans* entry experiments and the absence of a competitive component suggests that NDGA does not bind at the exofacial sugar‐binding sites of GLUT1. However, we also found that NDGA reduces *V*_max_ for *infinite‐cis* exit and increases the observed *K*_D_ for sugar interaction with the exofacial sugar‐binding site. This is a curious observation considering that resveratrol under the same kinetic schemes behaves as a noncompetitive agent on zero‐trans entry conditions and was unable to affect substrate affinity for the exofacial sugar‐binding site in infinite‐exit assays 15. There are two ways to interpret these results: (a) NDGA binds to an exofacial‐binding site which is independent from the substrate‐binding site, or (b) NDGA binds to an internal ligand‐binding site and exert a negative allosteric effect on ligand binding to the external sugar‐binding site.

This contrast between NDGA and resveratrol appears to be reminiscent of the action of methylxanthines over the activity of the GLUT1 carrier 10. The methylxanthines, pentoxifylline, caffeine, and theophilline are uncompetitive blockers of GLUT1‐mediated sugar uptake, but caffeine and theophylline decrease *V*_max_ for *infinite‐cis* sugar exit without affecting the affinity of the external sugar‐binding site for sugar. However, pentoxifylline, which contains a 5‐oxohexyl group in place of a methyl group at position 1 of the purine, reduces *V*_max_ for *infinite‐cis* exit and increases the apparent *K*_D_ for sugar interaction with the exofacial sugar‐binding site. We interpreted these results as consistent with an exofacial methylxanthine‐binding site, which was independent from the substrate‐binding site 10. As it has been argued that sidedness of action cannot be unambiguously determined by just kinetics experiments 27, 28, definitive characterization of the NDGA‐ and resveratrol‐binding site on GLUT1 should wait for either direct labeling studies or mutagenesis of putative interaction domains in combination with MD simulations.

Several studies have reported that NDGA targets cellular processes involved in cell proliferation and tumor growth in animal models. Here, we observed that NDGA decreases viability of proliferating leukemic cells in a dose‐dependent manner but does not alter the survival of normal nonproliferating white blood cells in culture. Our results are consistent with NDGA targeting one or more processes involved in cell growth, which are active only in highly proliferating cells. Thus, these results indicate that NDGA may be useful for cancer treatment, either alone or in combination with other drugs, because it affects only highly proliferating cells.

We found that NDGA is an effective inhibitor of sugar uptake through GLUT1 transporter on HL‐60 and U937 cells and also in human erythrocytes at micromolar concentrations, affecting substrate uptake in zero‐trans entry and exit assays. However, glucose uptake is hampered by NDGA with IC_50_ values 2–10 times higher (depending on the assay) than those observed for cell viability. These results may be explained by two reasons. First, we may have been unable to observe a correlation between glucose uptake and cell viability because of the different timescales to study both processes. While glucose uptake occurs in seconds, effects on cell viability can be only observed after hours of incubation. A second possibility is that NDGA, as a pleiotropic drug, has other targets whose inhibition/activation also results in decreased cell viability. For example, NDGA has been described as inhibitor of cell viability by affecting the lipooxygenase (LOX) and NADPH oxidase systems 29, 30. However, characterizing the effect of NDGA over other cell systems is outside the scope of this study and further studies will be needed to fully understand the effects of this pleiotropic drug.

The structurally similar polyphenols, NDGA, resveratrol, and quercetin have been described as cell sensitizers and glucose uptake inhibitors 11, 15, 31, 32. Because this may lead to combined therapies against cancer, it is important to determine the combined effect of these molecules on cell viability and glucose transport. Our future efforts will be focused on studying whether or not these polyphenols can simultaneously, and perhaps synergistically, affect glucose uptake and cell viability of leukemic cells.

## Author contributions

MS, AZ, AAP, and AMR were responsible for the conception and design of research; DL, DP, and LO, performed the experiments; MS, DL, AMR, and MV‐U analyzed data, drafted, edited and revised manuscript.
